# LTX-315 – a promising novel antitumor peptide and immunotherapeutic agent

**DOI:** 10.15698/cst2019.11.202

**Published:** 2019-11-03

**Authors:** Dagmar Zweytick

**Affiliations:** 1 Institute of Molecular Biosciences, Biophysics Division, University of Graz, Graz, Austria.; 2 BioTechMed-Graz, Graz, Austria.

**Keywords:** antitumor peptide, immunotherapeutic agent, melanoma, sarcoma

Host defense in mammals, as provided by the innate immune system, comprises proteins such as lactoferrin (LF), a multifunctional iron-binding glycoprotein original-ly discovered in bovine milk. LF is further pepsin-cleaved to a cationic amphipathic peptide, lactoferricin (LFcin; amino acid 1-45 of LF), which is known for its antimicro-bial, antiseptic, antiviral, antitumor and immunomodula-tory activities [1][2][3]. Bovine LFcin has been shown to inhibit liver and lung metastasis of both murine melano-mas and lymphomas [4] and to induce apoptosis in human leukemic and carcinoma cell lines [5][6]. LTX-315 [7] and LTX-302 [8], which derived of bovine LFcin by structural optimization, contain - amongst others - the non-coded residue β-diphenylalanine and show increased activity *in vivo* by peptide in-duced tumor regression and infiltration of the tumor by immune cells. LTX-315 is effective against multiple tumor types, and is therefore studied as novel immunothera-peutic agent in phase I/II clinical trials in combination with checkpoint inhibitors for treatment of advanced solid tumors, using the ability to reduce tumor growth and to induce de novo T-cell responses [9].

In the current issue of Cell Stress, Pittet and colleagues evaluated LTX-315 in conditional genetic mouse models of melanoma and sarcoma that are so far mainly resistant to standard treatment. Therefore, syngeneic grafts of murine melanoma B16F10, *Braf*- and *Pten*-driven melanoma as well as *Kras*- and *P53*-driven soft tis-sue sarcoma were studied in mice regarding their sensi-tivity towards LTX-315. These mutations are an ideal model, since they are often found in human patients suffering of these cancer types, as well are these tumor models, as also murine melanoma B16F10, poorly infil-trated by T cells and resistant to immune checkpoint therapy. The authors show a two-phase response in the tumor models triggered by the intratumoral injection with the peptide. The first phase of response is a rapid (within minutes) disruption of tumor vasculature and decrease of tumor burden. This direct antitumor effect seems to occur by induced cell lysis blocking the oxygen and nutrients supply by the tumor vasculature without the help of antitumor lymphocytes. The second phase of response is however as important for the antitumor (long-term) effect of the peptide. It endures over sever-al weeks and is characterized by a tumor infiltration with CD8+ T cells that is normally very poor in the described tumor models and can display antitumor functions. Fur-ther, immune cells such as CD4+ T cells and natural killer (NK) cells were shown to migrate into the tumor envi-ronment upon treatment with LTX-315. This effect of triggering an antitumor immune response was more pronounced in the melanoma than in the sarcoma mod-els, which might be due to the lower mutational load of the latter. However, this long-term conversion of a poor-ly to a highly immunogenic tumor promises a long-term antitumor immunity by prevention of tumor regrowth after treatment.

Malignant melanoma and fibrosarcoma both exhibit poor treatability and prognosis and therefore demand for new therapy, such as LTX-315 studied within the cur-rent issue by Pittet and colleagues. Depending on the progression, surgery, classical radio- and chemotherapy are applied for the treatment of malignant melanoma, though therapeutic options are limited due to metastasis and chemo-resistance [10]. In the last years, targeted- and immunotherapies (e.g. CTLA4-, PDL1/2- and BRAF inhibi-tors) have been developed and are promising to be more specific and exhibit less side effects [11]. However, these therapies also only yield limited improvement of survival and even here resistances are observed towards MAPK inhibitors (BRAF and MEK inhibitors) and immunothera-peutic drugs like CTLA4 inhibitors [12]. Fibrosarcoma is a rare but highly malignant soft tissue tumor with a low sensitivity towards radio- and chemotherapy, as well as a high rate of reoc-currence and therefore poor prognosis [13].

Thus, in urgent need of new antitumor therapies and based on the results that LTX-315 triggers CD8+ T cell accumulation in the mouse models, two melanoma and two sarcoma patients were treated with LTX-315 intra-tumorally. Biopsy evaluation by histology revealed pep-tide treatment induced CD3+ and CD8+ infiltration of both tumor types in all four patients, providing evidence for a promising use in clinical therapy against these can-cer types. The ability of the peptide to convert a "cold" into a "hot" (immunogen-ic) tumor further encourages a combinational therapy of LTX-315 with checkpoint inhibitors. A triple effect could be provoked by (i) direct disruption of the tumor by LTX-315, (ii) attack by immune cells induced by checkpoint inhibitors such as anti-CTLA4 and finally (iii) a stop of regrowth by peptide induced antitumor immunity.
